# Percutaneous leverage reduction with kirschner-wire fixation assisted by elbow arthrography for pediatric radial neck fractures

**DOI:** 10.3389/fped.2025.1571774

**Published:** 2025-09-19

**Authors:** Hai Jiang, Tao Li

**Affiliations:** Department of Pediatric Orthopedics, Northwest Women’s and Children’s Hospital, Xian, China

**Keywords:** children, radial neck, fracture, reduction, arthrography

## Abstract

**Objective:**

Radial neck fractures in children can easily become complicated if not managed properly. Percutaneous reduction using the leverage technique with or without internal fixation with a Kirschner wire (K-wire) is a minimally invasive approach for treating angulated radial neck fractures in children. The study aims to evaluate the radiological and clinical outcomes of percutaneous leverage reduction assisted by elbow arthrography for pediatric radial neck fractures.

**Methods:**

From January 2016 to June 2020, we treated 47 children with angulated radial neck fractures, including 35 boys and 12 girls. The patient's age ranged from 2 to 13 years, with an average age of 6 years 9 months. According to Judet classification, 21 cases were classified as Type III, 15 cases as Type IVa and 11 cases as Type IVb. To overcome the difficulty of reduction caused by the absence of the ossified radial head centers in young children, we used intraoperative elbow arthrography to assist with the reduction. After achieving satisfactory reduction, one or two K-wires were inserted percutaneously to fix the fracture site and prevent reduction loss.

**Results:**

All cases were followed up for an average of 43 months, ranging from 24 months to 90 months. No radial head necrosis or synostosis of the proximal ulna and radius was observed during the long-term follow-up. No epiphyseal arrest or valgus of the elbow was noted at the end of the follow-up. According to the Metaizeau reduction classification, 42 cases were rated excellent, and 5 cases as good. Based on the Metaizeau clinical classification, 45 cases were excellent and 2 were good.

**Conclusion:**

Closed reduction assisted by intraoperative elbow arthrography, combined with percutaneous leverage technique and internal fixation with K-wires, achieved satisfactory reduction and functional outcomes in children with angulated radial neck fractures, even in cases where the radial head ossification centers were not yet visible.

Level of Evidence: Level IV—observational study design.

## Introduction

Radial neck fractures in children can easily become complicated if not managed properly. Open reduction and internal fixation are commonly used to treat severe angulated radial neck fractures (1–3). However, complications such as scar formation, ischemic necrosis of the radial head, and limited elbow function are frequently observed (4–6). In recent years, minimally invasive surgical treatments for angulated radial neck fractures in children have been shown to reduce complications and improve postoperative function (7–9). Percutaneous reduction using the leverage technique, with or without internal fixation using K-wires, is a minimally invasive approach for treating these fractures (10–12). However, achieving proper reduction during minimally invasive surgery is technically challenging, especially in young children whose radial head ossification centers are not visible. Additionally, reduction loss often occurs after surgery (13). Stable internal fixation is crucial for preventing reduction loss.

To overcome the reduction difficulty caused by the absence of the ossified radial head centers in young children, we used intraoperative elbow arthrography to assist with percutaneous K-wire reduction using the leverage technique. For some older children, we also used arthrography to assist with the reduction though the ossified radial head centers were visible. After achieving satisfactory reduction, one or two K-wires were inserted percutaneously to fix the fracture site and prevent reduction loss.

## Methods

From January 2016 to June 2020, we treated 47 children with angulated radial neck fractures, including 35 boys and 12 girls. The patients’ ages ranged from 2 to 13 years, with an average age of 6 years 9 months. According to Judet classification,21 cases were classified as Type III, 15 cases as Type IVa and 11 cases as Type IVb (Table 1). The radial neck–shaft angle was measured as the angle between the line perpendicular to the articular surface of the radial head and the axis of the radius. Translation was measured as the distance in millimeters measured from the center of the radial head to the axis of the radius. The left side was affected in 29 cases, and the right side was affected in 18 cases. The ulnar olecranon fracture was concomitant in 15 cases. There were no vascular or nerve injuries.

**Table 1:** Judet classification of radial neck fractures, along with the number of patients in groups.

Judet classification number		Number of patients(%)
Type	Degree of displacement	
I	No displacement or horizontal shift of epiphysis	0
I	<30° angulation	0
III	30° a 60° angulation	21 (44.7%)
IVa	60° a 80° angulation	15 (31.9%)
IVb	>80° angulation	11 (23.4%)

**Figure 1:**
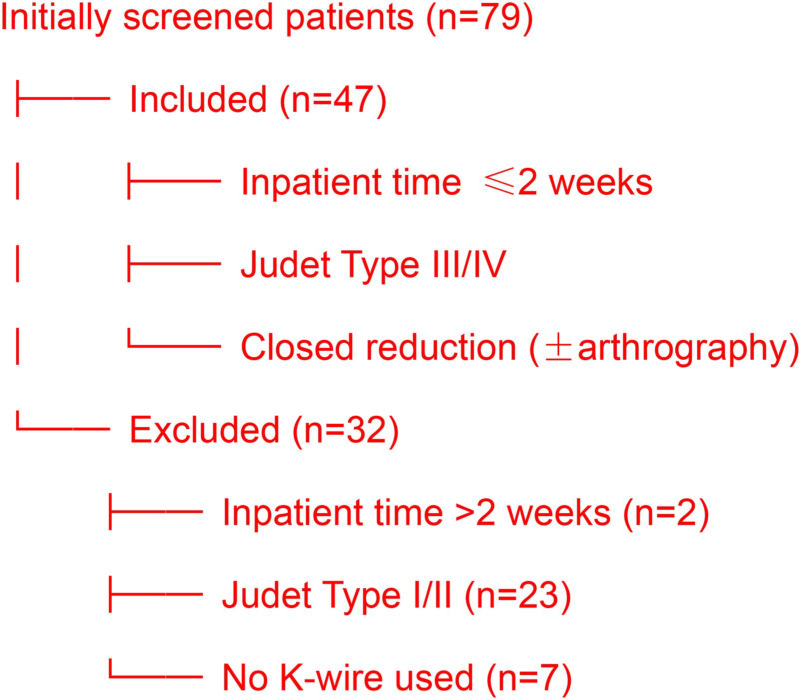
Patient selection flowchart.

The following patients were excluded from the study: (1) inpatient time from injury more than 2 weeks, (2) with Judet classification Type I or II, (3) closed reduction without Kirschner (Figure 1).

## Surgical technique

Intraoperative arthrography was performed using a lateral or posterior approach with iohexol. The needle was inserted anteromedially into the elbow joint at an angle of approximately 45°. Fluoroscopy was used to guide needle positioning and ensure proper placement. Proper placement was confirmed either by needle aspiration of a hematoma or by injecting a small amount of saline into the joint and observing the ease of backflow. The contrast agent used was iohexol. Iohexol was diluted with normal saline at a 1:1 ratio. The amount of iohexol injected was determined by the child's age: younger children (<3 years old) received 1.0 ml, while older children (>3 years old) received 1.5 ml.

The elbow was passively flexed and extended gently to ensure the even distribution of the contrast agent throughout the elbow joint. The displacement of radial neck fracture was clearly demonstrated through fluoroscopy.

We employed the leverage technique to reduce the fracture using a 2.0 mm K-wire. The K-wire was percutaneously inserted into the elbow joint, advancing from distal to proximal along the fracture line. By inserting the K-wire into the fracture site, we leveraged the fracture to achieve proper alignment. For these patients classified Judet Type IVb, the displacement and angulation were severe. We inserted the K-wire to push and leverage the radial head, to reduce the displacement and angulation. Through the step, these patients were changed to be Judet Type III or Type II. Then we repeated the leverage reduction with K-wire under the assistance of C-arm until a satisfied reduction was attained. This maneuver ensured that the radial head surface was horizontally aligned with the lateral condyle in the anteroposterior view. In the lateral projection, the articular surface of radial head should be perpendicular to the radial shaft and parallel to the humeral shaft. The reduction position was confirmed to be excellent by intraoperative fluoroscopy. The leverage K-wire was then further inserted into the opposite cortex of the proximal radius. In 38 patients, a single K-wire was used to treat the radial neck fracture. In nine additional patients, a second 1.2 mm or 1.5 mm K-wire was inserted percutaneously along the edge of the articular surface, extending from the fracture site to the opposite cortex. The external parts of the K-wires were bent and removed after internal fixation. All patients with ulnar olecranon fractures underwent closed reduction and percutaneous fixation with 2 or 3 K-wires.

Postoperatively, the elbow joint was immobilized with a cast in 90° of flexion and in a supinated forearm position for 3–4 weeks. The K-wire was typically removed 3–4 weeks after surgery. The combined internal fixation for olecranon fractures was usually removed 3–4 weeks postoperatively, depending on the fracture healing progress. Elbow joint function exercises could be initiated immediately after K-wire removal.

Two blinded orthopedic surgeons (Dr. Jiang, Dr. Li) independently measured angulation and translation in all cases. Intraclass correlation coefficient (ICC, two-way random-effects model) assessed inter-rater reliability.

## Results

All cases were followed up for an average of 43 months, ranging from 24 months to 90 months. Postoperative reduction was assessed using x-ray within one week after the operation. No loss of reduction during short-term follow-up, and there were no instances of infection or injury to the deep branch of the radial nerve. All the fractures achieved union within 4 weeks after operation. Long-term follow-up revealed no radial head necrosis or synostosis between the proximal ulna and radius. At the end of follow-up, no epiphyseal arrest or valgus deformity of the elbow was observed (Table 2). ICC for angulation and translation was 0.91 (95% CI: 0.86–0.95), indicating excellent agreement.

**Table 2:** Descriptive data of the radial neck-shaft angle.

Radiographic parameter	Phase	Statistical valuses			*P* value
		M	SD	95%CI	
Radial neck-shaft angle(°)	Preoperative	55.4	13.6	42.7–53.8	<0.001
	Postoperative	4.7	3.2	2.4–4.8	<0.001
	Last follow-up	0.6	1.3	n/0	
Translation(mm)	Preoperative	4.2	1.5	2.1–3.5	<0.001
	Postoperative	0.4	0.7	0.2–0.7	<0.001
	Last follow-up	0.05	0.2	n/0	

M, mean; ME median; SD standard deviation; CI confidence interval.

There were five cases with slight forward displacement and slight angulation of the radial head during intraoperative reduction, which were not further adjusted. At the end of follow-up, x-ray examination showed that the proximal radius was in good shape, with the displacement and angulation well corrected.

**Figure 2:**
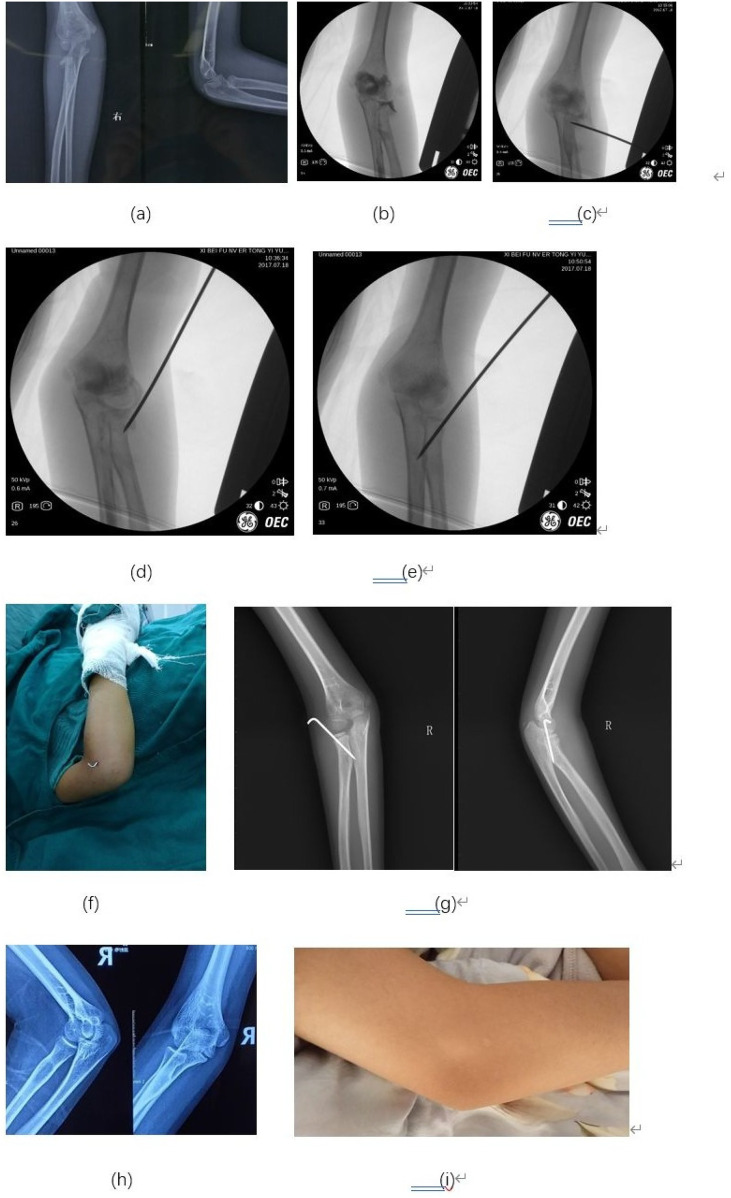
Typical case: An 8-year-old girl presented with a right elbow injury. **(a)** The preoperative x-ray showed 62° angulation of the radial neck fracture. **(b)** Arthrography provided clearer visualization of the radial neck angulation, more accurately delineating the fracture extent. **(c)** A 2.0 mm K-wire was inserted laterally and positioned distal to the fracture site. **(d)** Leverage reduction was performed using the K-wire to realign the fracture fragments. **(e)** The K-wire was further advanced to penetrate the cortex, providing additional stability to the reduced fracture. **(f)** The external portion of the K-wire was carefully removed, ensuring secure fixation while minimizing soft tissue irritation. **(g)** Anteroposterior (AP) and lateral projection (LP) x-rays taken one month postoperatively demonstrated successful union of the fracture. **(h)** AP and lateral x-rays at 7.5 years demonstrated excellent humeroradial and proximal radioulnar congruity, confirming long-term stability. **(i)** At 7.5 years, only a faint scar remains on the lateral elbow, yielding minimal cosmetic impact.

According to the Metaizeau reduction classification (14), 42 cases were rated as excellent, and five cases were rated as good (Table 3). Based on the Metaizeau clinical classification (15), 45 cases were excellent, and two cases were good. All the patients had a good function of the affected elbow (Table 4). The flexion and extension of the affected elbow was normal. There was no limitation of the pronation and supination function of the affected forearm. No patient needed to be operated again. A typical case with an excellent postoperative outcome is illustrated in Figures 2a–f.

**Table 3:** Radiological metaizeau classification after surgical treatment, along with the number of patients in groups.

Metaizeau classification		Number of patients (%)
Result	Reduction achieved	
Very good	Anatomic reduction	42 (89.36%)
Good	<20°	5 (10.64%)
Satisfactory	20–40°	(0%)
Poor	>40° 0	(0%)

**Table 4:** Descriptive of the ranges of motion of the elbow.

Rom	Upper limb	Statistical parameter			*P* value
		M	SD	95%CI	
Flexion(°)	Affected	135.5	3.6	135.1–139.6	<0.001
	Unaffected	140.7	0.5	139.7–140.4	
Extension(°)	Affected	0.8	1.6	0.0–0.7	>0.05
	Unaffected	0.0	0.0	n/o	
Supination(°)	Affected	80.4	10.7	74.2–83.5	<0.001
	Unaffected	85.2	3.8	83.7–87.4	
Pronation(°)	Affected	81.3	10.5	73.1–82.6	>0.05
	Unaffected	84.7	3.9	83.2–86.8	

M, mean; SD standard deviation; CI confidence interval.

## Discussion

Radial neck fractures are relatively uncommon in children, accounting for approximately 1% of all pediatric fractures. They are the third most common type of elbow joint fracture in children. Treating angulated fractures remains challenging, particularly in young children whose radial head ossification centers have not yet appeared. If not managed properly, several complications may arise, including avascular necrosis, early physeal closure, proximal radioulnar synostosis, cubitus valgus, and limited elbow function (16, 17). Closed reduction should be the first-line treatment for displaced and angulated fractures (13). Achieving a satisfactory, if not anatomic, reduction remains a challenge for pediatric orthopedic surgeons, especially in young children. For older children, the presence of a visible radial head ossification center on radiographs facilitates easier intraoperative reduction. However, in younger children, the absence of this ossification center complicates preoperative assessment of the direction and extent of displacement. Intraoperative arthrography can enhance the visualization of elbow joint structures, including the articular surfaces of the distal humerus and radial head (18). This technique allows for a more accurate understanding of the spatial changes in the fracture and guides the reduction process.

Successful reduction must be followed by appropriate internal fixation to prevent secondary displacement. Steinberg (19) reported that 22 out of 28 cases of fractures treated with plaster fixation alone after reduction experienced secondary displacement. Thus, proper internal fixation must be performed. Common internal fixation methods include elastic intramedullary nailing, K-wire intramedullary fixation, and percutaneous K-wire fixation (20–23). Indirect fixation using intramedullary K-wires has been found to be unstable. Eberl (24) used an intramedullary K-wire to treat 42 children with radial neck fractures. Loss of reduction occurred in 7 cases after the operation. In contrast, percutaneous K-wire fixation, as used in our cases, provides effective stabilization with a short lever arm, preventing postoperative displacement.

Elbow arthrography has been shown to be a useful diagnostic tool in pediatric elbow trauma (25–27). It has been successfully applied in the treatment of distal humerus epiphysis separation in children (28). Javed (18) reported a case of a four-year-old boy with a radial neck fracture and a non-ossified radial epiphysis, where arthrography facilitated the reduction process by clearly delineating the radial head and capitellum. This highlights the importance of intraoperative arthrography in achieving accurate reduction. Nowicki (29) frequently used arthrography to evaluate radial neck fractures of children before and after reduction.

Before proceeding with internal fixation, it is crucial to ensure that the reduction is adequate. The articular surface of the radial head should be parallel to the humeral head in the anteroposterior (AP) view and perpendicular to the radial shaft in the lateral projection (LP) view, with no anterior or posterior displacement. If the position is unsatisfactory, the direction of the K-wire should be adjusted under fluoroscopic guidance until optimal results are achieved. After one K-wire was fixed in the fracture site, we test the stability in pronation position. If the angulation or displacement increased in pronation position, it indicated unstable using one K-wire fixation. So we supinated the elbow and inserted another K-wire to fix the fracture site to confirm the stability of the fracture.

Percutaneous leverage reduction and fixation to treat pediatric radial neck fractures has been reported by Cossio et al. in 2014 (30). They used only a single K-wire to perform percutaneous reduction and internal fixation in one-step. Most patients achieved excellent x-ray results and excellent clinical results after using this method. The mean age was 9.1 years. The radial head ossification center on radiographs was visible in this age group. In our group, many patients were younger than 6 years. The radial head ossification center on radiographs was not visible in younger patients. So we added intraoperative arthrography to make the articular surfaces of the distal humerus and radial head visible before the leverage reduction process. And with the assistance of arthrography, the reduction became easier. There were several modified percutaneous leverage technique for pediatric radial neck fracture reduction reported by other authors (10, 31). They used K-wire intramedullary to fix the radial neck. All the clinical results were satisfied.

Several considerations should be kept in mind. First, the insertion site and leverage direction must be carefully chosen to avoid injury to the deep branch of the radial nerve. This can be achieved by entering the needle through the safe zone, which is located at the proximal end of the radius in the coronal plane. Pronation of the forearm during K-wire insertion increases the distance between the deep branch of the radial nerve and the radial head, reducing the risk of injury. Second, slight residual angulation and displacement after reduction can often be remodeled later; therefore, repeated reduction attempts should be avoided to minimize tissue injury. Third, the K-wire must be carefully selected, with a maximum diameter of 2.0 mm, and must pass through the internal cortex of the radial neck to ensure stability.

Elastic stable intramedullary nailing (ESIN) is currently the more widely adopted approach for pediatric radial neck fractures. Compared with ESIN internal fixation, there are several advantages in the k-wire leverage reduction method. The advantages of our method include minimal trauma, avoidance of local soft tissue injury from repeated manipulation, no surgical scars, no need for secondary hospitalization for implant removal, and a lower economic burden on families. However, the primary limitations of this study are the relatively small sample size and the retrospective study design. Additionally, the results are based on a single technique without a control group for comparison. Future studies should aim to address these limitations through larger, prospective, and comparative designs.

## Conclusions

Closed reduction assisted by intraoperative elbow arthrography, combined with percutaneous leverage technique and internal fixation with K-wires, achieved satisfactory reduction and functional outcomes in children with angulated radial neck fractures, even in cases where the radial head ossification centers were not yet visible.

## Data Availability

The original contributions presented in the study are included in the article/Supplementary Material, further inquiries can be directed to the corresponding author.
